# Development, Validation, and Assessment of Clinical Impact of Real-time Alerts to Detect Inpatient As-Needed Opioid Orders With Duplicate Indications: Prospective Study

**DOI:** 10.2196/28235

**Published:** 2021-10-25

**Authors:** Elsie Rizk, Joshua T Swan

**Affiliations:** 1 Department of Pharmacy Houston Methodist Houston, TX United States; 2 Department of Surgery Houston Methodist Houston, TX United States

**Keywords:** opioid stewardship, pain, as-needed opioids, duplicate orders, automated alerts

## Abstract

**Background:**

As-needed (PRN) opioid orders with duplicate indications can lead to medication errors and opioid-related adverse drug events.

**Objective:**

The objective of our study was to build and validate real-time alerts that detect duplicate PRN opioid orders and assist clinicians in optimizing the safety of opioid orders.

**Methods:**

This single-center, prospective study used an iterative, 3-step process to refine alert performance by advancing from small sample evaluations of positive predictive values (PPVs) (step 1) through intensive evaluations of accuracy (step 2) to evaluations of clinical impact (step 3). Validation cohorts were randomly sampled from eligible patients for each step.

**Results:**

During step 1, the PPV was 100% (one-sided, 97.5% CI 70%-100%) for moderate and severe pain alerts. During step 2, duplication of 1 or more PRN opioid orders was identified for 17% (34/201; 95% CI, 12%-23%) of patients during chart review. This bundle of alerts showed 94% sensitivity (95% CI 80%-99%) and 96% specificity (95% CI 92%-98%) for identifying patients who had duplicate PRN opioid orders. During step 3, at least 1 intervention was made to the medication profile for 77% (46/60; 95% CI 64%-87%) of patients, and at least 1 inappropriate duplicate PRN opioid order was discontinued for 53% (32/60; 95% CI 40%-66%) of patients.

**Conclusions:**

The bundle of alerts developed in this study was validated against chart review by a pharmacist and identified patients who benefited from medication safety interventions to optimize PRN opioid orders.

## Introduction

Duplicate as-needed (PRN) opioid orders that are indicated for the same pain level can lead to medication errors, opioid-related adverse drug events, and confusion among bedside nurses. Hospital accreditation standards and pain management guidelines recommend constructing mutually exclusive pain levels for PRN opioid orders to establish clear indications and avoid therapeutic duplication of pain scales for PRN indications [1-4]. The proportion of patients having multiple PRN opioid orders with a duplicate indication was among the critical quality indicators established for an Opioid Stewardship Program across a multihospital health system [5]. Therefore, there is a need to develop clinical decision support and automated processes to optimize this high-priority quality indicator.

Epic is an electronic health record (EHR) system with one of the largest market shares among hospitals in the United States; however, the current functionality of Epic does not allow for triggering drug-drug interactions or best practice alerts based on the PRN indication field specified in medication orders [6]. The purpose of this project was to build and validate real-time alerts within a third-party pharmacovigilance software that detect PRN opioid orders with a duplicate pain indication and provide clinicians with a clinically impactful tool to optimize the safety of PRN opioid orders in the hospital EHR.

## Methods

### Study Description

This was a prospective, program development study. Several study activities were observational and aimed to detect medication safety events in the EHR. One study activity was interventional where a clinical pharmacist improved patient care using real-time alerts in accordance with a new hospital policy. The hospital’s Institutional Review Board approved this study with a waiver of informed consent.

The Houston Methodist Opioid Stewardship Program collaborated with VigiLanz Corporation, a third-party pharmacovigilance software company that receives real-time data from the hospital’s EHR and creates condition-based alerts that identify specific patient situations or medical events. For example, if a new order for warfarin was placed in the EHR without any international normalized ratio values in the previous 24 hours, an alert would be generated to notify the pharmacist to initiate appropriate therapy monitoring interventions. Other areas of alerting include drug interactions, lab monitoring, antibiotic culture mismatch, medication dosage adjustments, treatment-related adverse events, and other therapy elements [7].

A set of VigiLanz alerts was created to detect patients with 2 or more PRN opioid orders with duplicate pain category indications. The hospital EHR that was used across the Houston Methodist health system to send data to VigiLanz and inform the alerts was Epic 2018 (Epic Systems Corporation).

### Best Practices for Medication Safety

Through interprofessional stakeholder engagement among pharmacists, nurses, physicians, and medication safety specialists, the Houston Methodist Opioid Stewardship Program established best practices for inpatient prescribing of PRN opioid orders to reduce the risk of medication errors. Unless clear parameters are provided, multiple PRN opioid orders for the same pain level are inappropriate and may cause confusion within the care team and lead to duplicate opioid administration. Therefore, the Opioid Stewardship Program created a schema to categorize multiple PRN opioid orders for the same pain level as appropriate or inappropriate using the following medication order attributes: (1) route and formulation, (2) use of the linked order group functionality in Epic, and (3) clear administration instructions. For example, a patient might have a PRN order for oral oxycodone for severe pain along with intravenous morphine PRN for severe pain to be administered as an alternative if the patient cannot tolerate oral intake. When duplicate PRN opioid orders for the same pain level are necessary, clear administration instructions should be provided to the bedside nurse to guide selection of the appropriate medication, and orders may be placed in a linked order group. In Epic, orders that are linked using the “OR” linking logic provide a safety enhancement feature that would notify nurses about the risk of duplicate administration if one of the orders was about to be administered too soon following the administration of another order in the linked group. To empower clinical pharmacists to resolve duplicate PRN opioid orders using their clinical judgment, the Opioid Stewardship Program developed a hospital policy that organized duplications into 5 categories and authorized pharmacists to provide specific interventions for each category, as shown in Table 1.

**Table 1:** Categorization of multiple as-needed opioid orders as appropriate or inappropriate.

Category	Collected information			Evaluation of data	
	Same route and formulation	Clear administration instructions	Linked orders	Appropriate duplication	Potential medication safety intervention
1	No^a^	Yes	Yes	Yes	No change needed
2	No^a^	No	Yes	No	Clarify administration instructions^b^
3	No^a^	Yes	No	No	May add orders to a linked order group using the “OR” linking logic
4	No^a^	No	No	No	Clarify administration instructions and may link orders^b^
5	Yes	Yes or No	Yes or No	No	Discontinue all but one of the duplicate orders

^a^Two orders with the same route (eg, oral) are allowed as long as the orders have different formulations (eg, oral tablet and oral liquid) with clear administration instructions and use the “link” functionality in Epic for enhanced administration safety.

^b^Examples of administration instructions were suggested based on the medication formulation or route as follows: For oral tablets, the instruction was “Give if patient can receive oral tablet medication.” For oral solutions, the instruction was “Give if patient cannot receive oral tablet medication but can receive oral solution medication.” For intravenous injections, the instruction was “Give if patient is not able to receive oral medication.”

### Logic to Trigger Real-Time Alerts

Houston Methodist used a Health Level 7 (HL7) message interface to transmit medication order data from the EHR to the third-party pharmacovigilance software in real time. The relevant medication order variables included in this HL7 message were the medication name, route, frequency, dose, administration instructions, start date and time, and PRN indication. The order linking information in Epic was not included in the HL7 message and was not used by the alerts to categorize multiple PRN opioid orders as appropriate or inappropriate. Investigators developed operational definitions for types of duplications and then created a bundle of alerts to identify each of the operational definitions given in Table 2. Duplicate PRN opioid orders were classified into explicit duplicates that specified pain levels as mild, moderate, or severe and implicit duplicates where at least one of the duplicate orders had unclear PRN instructions that did not specify the pain scale range. Alerts were censored for opioid orders that were verified while a patient was in an operating room or a procedural area due to the temporary nature and unique medical record workflow of these opioid orders. To reduce alert fatigue for end users, each alert was set to create no more than 1 activation every 24 hours for the same patient.

**Table 2:** Classification of duplicate as-needed opioid orders.

Duplicates			VigiLanz alert name		Example
**Explicit duplicates**					
	2 or more PRN^a^ opioid orders for mild pain	Opioids PRN for mild pain		Tramadol 50 mg PO^b^ every 8 hours PRN for mild pain (score 1-3) AND acetaminophen-codeine 300-30 mg PO every 4 hours PRN for mild pain (score 1-3)	
	2 or more PRN opioid orders for moderate pain	Opioids PRN for moderate pain		Hydrocodone-acetaminophen 5-325 mg PO every 6 hours PRN for moderate pain (score 4-6) AND tramadol 50 mg PO every 6 hours PRN for moderate pain (score 4-6)	
	2 or more PRN opioid orders for severe pain	Opioids PRN for severe pain		Hydromorphone 1 mg IV^c^ every 4 hours PRN for severe pain (score 7-10) AND fentanyl 25 mcg IV every 3 hours PRN for severe pain (score 7-10)	
**Implicit duplicates**					
	2 or more PRN opioid orders where at least one order has unclear PRN instructions^d^	Opioids PRN for pain		Hydrocodone-acetaminophen 10-325 mg PO every 6 hours PRN for pain AND morphine 15 mg PO every 6 hours PRN for severe pain (score 7-10)	
	An opioid PCA^e^ order and another IV PRN opioid ordered separately^f^	Opioid PRN PCA		Hydromorphone IV PCA AND morphine 2 mg IV every 4 hours PRN for severe pain (score 7-10)	

^a^PRN: as-needed.

^b^PO: oral.

^c^IV: intravenous.

^d^The PRN instructions did not specify the pain scale range.

^e^PCA: patient-controlled analgesia.

^f^This includes orders for any pain level (mild, moderate, or severe).

### 3-Step Process for Specifying, Refining, and Validating Real-Time Alerts

**Figure 1:**
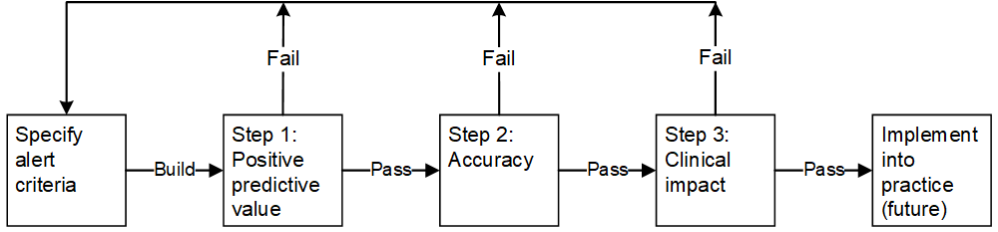
Alert development process. This 3-step process efficiently pilot-tested, revised, and validated bundles of alerts to detect duplicate as-needed opioid orders.

The investigators designed and implemented a 3-step process to develop a bundle of real-time alerts, validate their accuracy, and evaluate their clinical impact when used by pharmacists to identify duplicate PRN opioid orders, as indicated in Figure 1. This process was designed to quickly identify and resolve errors in alert performance by advancing from small sample, positive predictive value (PPV) evaluations (step 1) to intensive accuracy evaluations (step 2) and clinical impact evaluations (step 3). As medical record alerts commonly generate false positives that contribute to alert fatigue, our process measured and optimized the PPV as the first step [8]. After each step, the investigators would determine if the bundle of alerts needed further configuration (fail) or could be advanced to the next step of testing (pass). Results for the final bundle of alerts that passed all 3 steps are reported in this paper.

#### Step 1: Evaluation of PPV

On September 4, 2018, 10 active alerts for duplicate PRN opioid orders for each of the 2 most common types of alerts (moderate and severe pain) were randomly sampled from alerts generated from August 31, 2018 to September 4, 2018. Alerts were validated against active medication orders on the patients’ profiles by a pharmacist. Alerts identifying duplicate orders that were confirmed by the pharmacist’s chart review were categorized as true positive, and alerts that were not confirmed were categorized as false positive. The PPV was calculated by dividing the number of true positive alerts by the total number of alerts. The sample size of 10 was chosen to quickly identify and resolve errors in alert performance.

#### Step 2: Evaluation of Accuracy

A cross-sectional analysis of patients hospitalized on or after August 31, 2018, was conducted over 5 consecutive weekdays (September 6, 2018 until September 12, 2018). Patients admitted to acute care floors or intensive care units were randomly sampled once per day, and a patient could not be sampled more than once. Operating rooms, postanesthesia care units, catheterization labs, and other procedural areas were excluded from sampling. A pharmacist and a pharmacy intern conducted chart review to collect active PRN opioid names, doses, routes, frequencies, and PRN indications for each included patient. Alert-specific data were collected, including alert names, triggering opioid orders, and start and end dates of the triggering orders. As a patient may have more than one set of duplicate PRN opioid orders or more than one alert, this analysis was conducted at the patient level to match the workflow of medication profile review by clinicians.

It is recommended that medical record alerts be evaluated like diagnostic tests by calculating the performance characteristics of accuracy, sensitivity, specificity, PPV, and negative predictive value (NPV) [8]. Duplicate PRN opioid orders identified through this chart review served as the reference standard for this validation of duplicate opioid alerts to identify true and false positives and negatives. Accuracy was the number of patients correctly classified by the alerts (true positives plus true negatives) divided by the total number of patients evaluated. Sensitivity was the proportion of patients with duplicate PRN opioid orders who had an alert. Specificity was the proportion of patients without duplicate PRN opioid orders who did not have an alert. PPV was the proportion of patients with an alert who had duplicate PRN opioid orders. NPV was the proportion of patients with no alerts who did not have duplicate PRN opioid orders.

#### Step 3: Evaluation of Clinical Impact and Face Validity

Step 3 included 60 patients identified from a random sample of patients with an alert on a specific calendar day (or the previous calendar day) over 6 nonconsecutive days in September and October 2018, and a patient could not be sampled more than once. A pharmacist conducted medication profile review in the EHR to optimize the PRN opioid orders in accordance with the algorithm described in Table 1 through the following actions: (1) discontinuing one or more duplicate PRN opioid orders, (2) clarifying order administration instructions, (3) contacting the prescriber or the bedside nurse, (4) changing the pain level indication, and (5) assigning PRN opioid orders to linked order groups in the medical record to prevent duplicate administration of orders with different routes or formulations. Whenever PRN opioid orders were discontinued, the pharmacist documented the rationale for discontinuation (orders with older start dates, no recent administration, documented adverse events, or no associated order set). For each patient, the pharmacist recorded a start time when the alerts and patient’s medical record were opened to evaluate active PRN opioid orders. The pharmacist then recorded an end time after completing all necessary actions and documenting interventions in the pharmacovigilance software. The time in minutes between each start and end time was calculated. To estimate the time effort for future implementation of this program into routine practice for clinical pharmacists and other health care professionals, linear regression was conducted to estimate the time needed by the pharmacist to conduct chart review and resolve duplicate PRN opioid orders based on the extent of actions taken.

## Results

### Step 1: Evaluation of PPV

During the study period for step 1, 9% (10/114) of moderate pain alerts and 14% (10/71) of severe pain alerts were randomly sampled and evaluated. The PPV was 100% (one-sided, 97.5% CI 70%-100%) for moderate alerts and 100% (one-sided, 97.5% CI 70%-100%) for severe alerts. The investigators interpreted this as success and advanced to step 2.

### Step 2: Evaluation of Accuracy

During the study period for step 2, 30% (201/662) of eligible patients were randomly sampled and had 241 active PRN opioid orders, as observed in Table 3. Chart review identified 1 or more PRN opioid order duplications for 17% (34/201; 95% CI, 12%-23%) of patients. Of these 34 patients, duplication was identified for the moderate pain scale in 12 (35%), severe pain scale in 9 (26%), both moderate and severe pain scales in 7 (21%), and unclear PRN instructions that did not specify the pain scale range in 6 (18%). This bundle of alerts showed high sensitivity (94%) and specificity (96%) for identifying patients who had duplicate PRN opioid orders, as indicated in Tables 4 and 5. Investigators interpreted this as success and advanced to step 3. On September 18, 2018 (after step 2 was completed), 2 implicit alerts were added to the bundle to identify duplication between orders with unclear PRN indications (“breakthrough pain” or “any pain”) and any other PRN opioid order.

**Table 3:** Prevalence of active as-needed opioid orders in step 2 (N=241).

PRN^a^ indication pain category	Pain score	Frequency, n (%)
Mild	1-3	3 (1)
Moderate	4-6	123 (51)
Severe	7-10	93 (39)
Mild and moderate	1-6	2 (1)
Moderate and severe	4-10	6 (2)
Patient-controlled analgesia	Not specified	5 (2)
Unspecified pain category	Not specified	9 (4)

^a^PRN: as-needed.

**Table 4:** Comparison of chart review reference standard with the bundle of alerts in step 2 (N=201).

Alert fired	Reference standard		
	Active duplicate orders	No active duplicate orders	Total
Alert	32 (true positive)	7 (false positive)	39
No alert	2 (false negative)	160 (true negative)	162
Total	34	167	201

**Table 5:** Performance characteristics of the bundle of alerts in step 2 (N=201).

Performance characteristics	Value (%)	95% CI (%)
Sensitivity	94	80-99
Specificity	96	92-98
PPV^a^	82	67-93
NPV^b^	99	96-100
Accuracy	96	92-98

^a^PPV: positive predictive value.

^b^NPV: negative predictive value.

### Step 3: Evaluation of Clinical Impact and Face Validity

**Figure 2:**
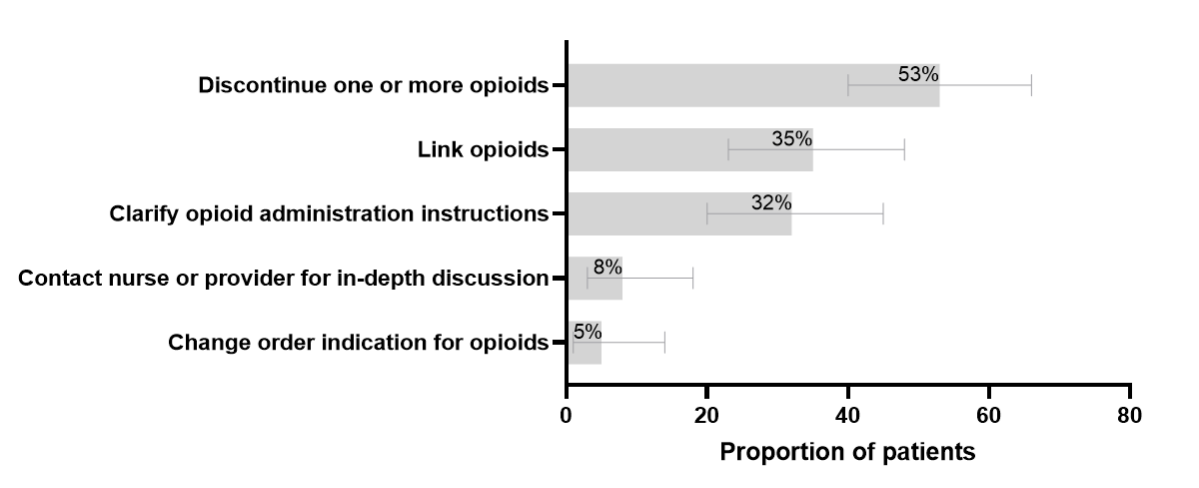
Clinical impact assessment in step 3 (60 patients). The categories are not mutually exclusive.

**Figure 3:**
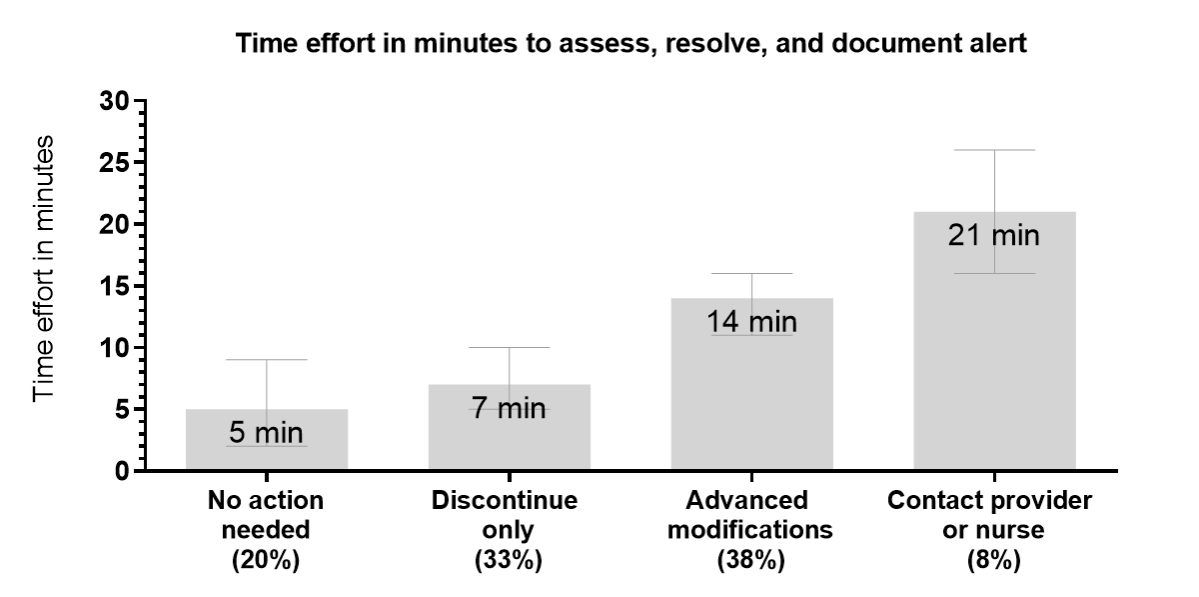
Time effort needed by the pharmacist to assess and resolve duplicate as-needed opioid alerts and document interventions in step 3 (60 patients). The average time effort and 95% CI were calculated by conducting linear regression using the following mutually exclusive categories of actions taken for each patient: (1) no action needed, (2) 1 or more orders discontinued, (3) advanced modifications performed for at least 1order (linking orders, clarifying administration instructions, or changing the PRN (as-needed) indication, and (4) provider or nurse contacted with or without other order modifications. These time estimates also include the time for study data collection, which may require slightly more time (1 or 2 minutes per patient) than routine documentation of clinical interventions by hospital pharmacists.

During the study period of 6 nonconsecutive days for step 3, 12% of the eligible patients (60/481) were randomly sampled, who accounted for 12% of the unique eligible alerts (79/678). A pharmacist reviewed the charts of all the patients. At least 1 intervention was made to the medication profile for 77% (46/60; 95% CI 64%-87%) of patients, which was interpreted by investigators as having a meaningful clinical impact and face validity. The most common actions taken for these 60 patients were discontinuing inappropriate duplicate PRN opioid orders (32, 53%), linking PRN opioid orders (21, 35%), and clarifying administration instructions (19, 32%), as shown in Figure 2. Using linear regression estimates, the average time needed by the pharmacist to assess alerts, resolve issues, and document interventions was 5 minutes (95% CI 2-9 minutes) if no action was needed, 7 minutes (95% CI 5-10 minutes) if 1 or more orders were discontinued, 14 minutes (95% CI 11-16 minutes) if advanced modifications were performed for at least 1 order (linking orders, clarifying administration instructions, or changing the PRN indication), and 21 minutes (95% CI 16-26 minutes) if a provider or nurse was contacted with or without other order modifications, as shown in Figure 3. The investigators interpreted this as success and advanced the bundle of alerts for future implementation into the workflow of hospital pharmacists.

## Discussion

### Principal Findings

Decreasing the proportion of hospitalized patients who have PRN opioid orders with duplicate pain level indications was identified as a top priority for opioid stewardship in hospitals and health care systems [5]. This study evaluated the accuracy, validity, and clinical impact of a set of real-time alerts to identify and address duplicate PRN opioid orders. In this 3-step process, step 1 allowed rapid assessment and revision of alerts, step 2 validated a bundle of alerts (sensitivity of 94% and specificity of 96%), and step 3 determined that a clinical pharmacist could make at least 1 intervention for 77% of patients with an alert. In a health system where pharmacists were authorized to optimize PRN opioid orders per hospital policy, the pharmacist spent 7 minutes or less per patient for half of the patients evaluated and resolved most alerts within 14 minutes. This 3-step process could be extended beyond pain intensity scales to develop a bundle of alerts that can identify duplicate analgesic PRN orders based on a patient’s functional status or duplicate PRN orders for other clinical indications, such as nausea or vomiting and gut motility.

### Limitations

These alerts were validated using a single EHR system (Epic) and would need additional evaluation in other EHR systems. The clinical impact observed in step 3 was evaluated at a single academic medical center, which may not represent the potential clinical impact at other hospitals with different clinical workflows and patient populations.

Although the linking orders feature provides additional safeguards, the current process for linking 2 or more active orders in Epic requires discontinuation of all relevant orders and substitution with new linked orders. As this workflow may increase the risk of medication errors, multiple hospital committees decided that linking of active orders could be optional if clear administration instructions were provided. When this study was conducted, linked orders were not commonly used, and data feeds from the EHR to the pharmacovigilance software did not include information on order linking. However, if linked orders are used more commonly in the future, this lack of information in the data feed could increase the frequency of false positive alerts, which are estimated to require a pharmacist review time of 5 minutes.

### Considerations for Clinical Implementation

The developed bundle of alerts can be used to track each occurrence of duplicate orders (event-based) or identify patient charts that need medication review (patient-based). If used to track each occurrence, these alerts need to be generated every time a duplication occurs (eg, every pair of duplicate orders). However, this tracking approach can generate multiple alerts for each patient daily and cause alert fatigue when identifying patients for medication review. One strategy to prevent alert fatigue is to apply censoring logic to the bundle of alerts so that the bundle will only generate 1 alert in a 24-hour period for each patient, regardless of how many duplicate orders were signed during the 24-hour period. When identifying patients for medication review, alerts can be communicated to pharmacists via automated emails, pages, or an alert queue (internet-based web page).

### Conclusions

The bundle of alerts developed in this study was validated against chart review by a pharmacist and identified patients who benefited from medication safety interventions to optimize PRN opioid orders. These alerts provide a real-time automated screening process that replaces intensive chart review needed to identify patients, thus allowing health care team members to spend more time optimizing orders that are unclear and potentially not safe. Our algorithm, which matches categories of inappropriate duplicate PRN opioid orders to potential medication safety interventions, can be used to develop policies that expand the scope of practice for clinicians at other institutions. Further research is needed to evaluate the impact of implementing these alerts into quality surveillance or clinical workflow on the frequency of hospitalized patients who have duplicate PRN opioid orders, which is an important medication safety issue.

## Abbreviations

EHR: electronic health record
HL7: Health level seven
NPV: negative predictive value
PPV: positive predictive value
PRN: as-needed
